# Nanomaterial Delivery Vehicles for the Development of Neoantigen Tumor Vaccines for Personalized Treatment

**DOI:** 10.3390/molecules29071462

**Published:** 2024-03-25

**Authors:** Xiaoyu Huang, Xiaolong Zhu, Huan Yang, Qinyi Li, Lizhi Gai, Xinbing Sui, Hua Lu, Jiao Feng

**Affiliations:** 1 School of Pharmacy, Hangzhou Normal University, Hangzhou 311121, China; xyhuang0701@163.com (X.H.); 2023112025090@stu.hznu.edu.cn (X.Z.); yhnpc1029@163.com (H.Y.); liqinyi2000620@163.com (Q.L.); hzzju@hznu.edu.cn (X.S.); 2 College of Material, Chemistry and Chemical Engineering, Key Laboratory of Organosilicon Chemistry and Material Technology of Ministry of Education, and Key Laboratory of Organosilicon Material Technology of Zhejiang Province, Hangzhou Normal University, Hangzhou 311121, China; lizhigai@hznu.edu.cn

**Keywords:** tumor vaccines, neoantigen, nanomaterials, vaccine delivery, personalized treatment

## Abstract

Tumor vaccines have been considered a promising therapeutic approach for treating cancer in recent years. With the development of sequencing technologies, tumor vaccines based on neoantigens or genomes specifically expressed in tumor cells, mainly in the form of peptides, nucleic acids, and dendritic cells, are beginning to receive widespread attention. Therefore, in this review, we have introduced different forms of neoantigen vaccines and discussed the development of these vaccines in treating cancer. Furthermore, neoantigen vaccines are influenced by factors such as antigen stability, weak immunogenicity, and biosafety in addition to sequencing technology. Hence, the biological nanomaterials, polymeric nanomaterials, inorganic nanomaterials, etc., used as vaccine carriers are principally summarized here, which may contribute to the design of neoantigen vaccines for improved stability and better efficacy.

## 1. Introduction

Cancer is a genomic disease characterized by genomic instability, accumulation of mutations, and structural alterations as tumors progress. The tumor antigens produced by these genomic variants can be recognized as non-autoantigens and trigger a cellular immune response. Cancer paradigms of many cancers harness the body’s immune system to attach to tumor cells. Immunotherapies have transformed the treatment modalities of cancers. Therapies such as adoptive therapy and immune checkpoint inhibitors (ICIs) have made impressive progress. However, their effectiveness varies throughout persons and tumor types. The efficiency of ICIs varies between 10% and 30% in solid tumors, and they can develop drug resistance and immune-related adverse events [1]. This could be because there was insufficient cytotoxic CD8^+^ T cell response present beforehand. Therefore, therapies that enhance antitumor immunity, such as anticancer vaccinations, represent a viable and promising treatment option.

Tumor-associated antigens (TAAs) are highly expressed on cancer cells, while tumor-specific antigens (TSAs) or tumor neoantigens are abnormal antigens that are only expressed on cancer cells and are recognized by the immune system [2]. Compared to TAA-based tumor vaccines, TSA-based vaccines have stronger and sustained immunogenicity and specificity, effectively avoiding inadvertent injury to nontumor sites, which are not affected by central tolerance, and reducing organismal tolerance [3]. In recent years, by developing technologies such as mass spectrometry (MS), next-generation sequencing (NGS) [4], and RNA sequencing [5], the identification and knowledge of tumor gene profiles, as well as the detection of mutations and prediction of potential epitopes of genes [6], have been enhanced, which has been instrumental in the further development of immunotherapy for cancer. However, several requirements, including immunization and the production of antigen-specific antibodies, must be met to trigger an effective systemic vaccination against malignancies. To ensure sufficient immune cell infiltration and tumor clearance, dendritic cells and functionally activated effector T cell subtypes must mediate a response in addition to the vital presence of pertinent tumor antigens, strong adjuvants, and related danger signals. Cancer vaccines include antigenic proteins, peptides, RNA/DNA, DCs loaded with cancer antigens, and whole tumor cell vaccines. Diverse nanocarriers were used to protect the delicate protein/peptide and RNA/DNA antigens and serve as adjuvants to boost cross-presentation to trigger CTLs. In this review, we will briefly introduce tumor neoantigen vaccines and the latest developments in nanotechnology for cancer vaccine delivery.

## 2. Tumor Neoantigen Vaccines

**Figure 1:**
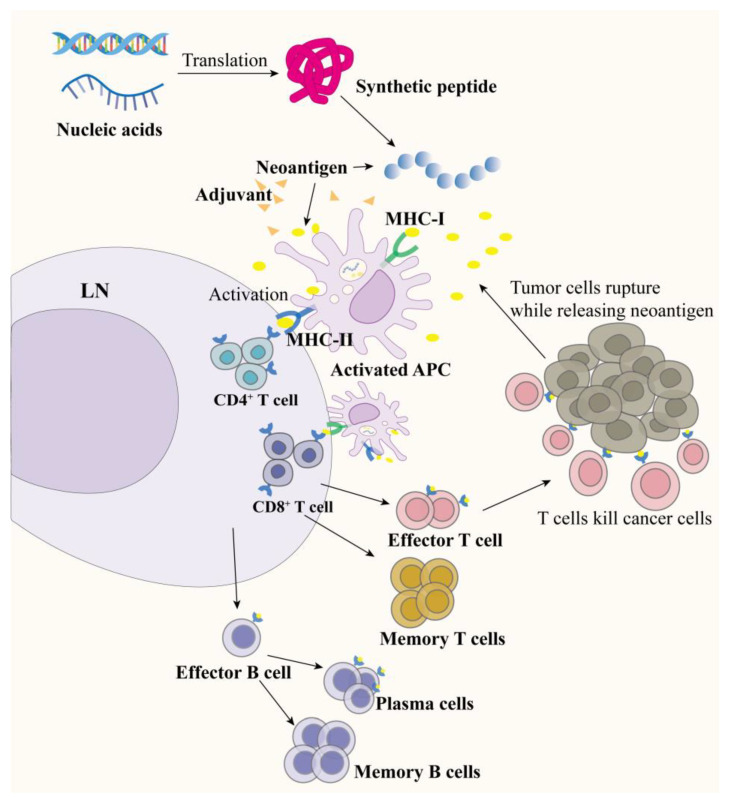
The mechanism of action played by vaccines: following neoantigen vaccination, APCs are stimulated to activate and subsequently induce potent CD4^+^/8^+^ T cells while generating memory cells capable of sustained tumor cell killing, and the fragmented tumor cells, in turn, continue to release neoantigens in vivo.

Vaccine design is also a critical component in inducing an efficient immune response in addition to predicting and characterizing the neoantigen [7]. The antigen has to have a suitable delivery platform and be able to be supplemented with a sensible adjuvant, which in turn stimulates antigen-presenting cells (APCs) to process, present, and induce a robust CD4^+^/8^+^ T cell response [8,9] (Figure 1). Current neoantigen vaccines mainly can be classified into dendritic cell (DC) vaccines, peptide vaccines, nucleic acid vaccines, and whole tumor cell vaccines [10,11].

### 2.1. Dendritic Cell Vaccines

Dendritic cells, first discovered by the Canadian scientist Steinman in 1973, are the most functional specialized APCs in the organism and play an important role in regulation, maintaining innate immune responses, and inducing adaptive immune responses [12]. The modified dendritic cells can express neoantigens and co-stimulatory molecules, which are processed and then presented to T cells to activate immune responses. However, in vitro expansion of DCs is more complex and costly, and the number of DCs required for vaccine production is large. To combat these issues, researchers have proposed the use of cell enrichment methods, sorting of fluorescently labeled cells, differentiation, and isolation from monocytes or leukocytes to improve the generation of isolated DCs [13]. Neoantigens can be transfected, pulsed, or electroporated onto DCs [14,15,16].

Carreno et al. first treated cancer patients with cancer neoantigen DC vaccines, which had previously been unrecognized human leukocyte antigen (HLA) class I-restricted neoantigens [17]. Through genomic analysis of tumor tissue samples from melanoma patients, functionally mature autologous interleukin (IL)-12p70-producing DC vaccines were subsequently utilized. In vivo, injection enhanced the specific recognition and response of T cells to neoantigens such as TMEM48 F169 L, SEC24 A P469 L, and others. Vaccination significantly increased the frequency of TCRβ clonotypes and expanded the diverse neoantigen TCR repertoire.

In another study, the stimulating factor CAIX was found to be delivered to autologous dendritic cells via adenovirus (Ad), supplemented by the immune-enhancing cytokine granulocyte-macrophage colony-stimulating factor (GM-CSF). Vaccination triggers a specific response against renal cell carcinoma (RCC) expressing the CAIX antigen. Combining ICIs weakened tumor immunosuppression to enhance vaccine efficacy. However, this study also reflects DC vaccines’ limitations in weak immune activation and the need for repeated vaccinations [18].

In addition to the above, the immunosuppressive environment in vivo and factors such as low specific T cell affinity, weak targeting ability, and tumor microenvironment can affect the effectiveness of DC vaccines [19,20]. The practical capability of homing to the lymph nodes and efficiency of antibody release from DCs are also limitations of DC vaccines, so the objective tumor response rate rarely exceeds 15% in clinical response [21].

In addition to having an important role in antitumor immunotherapy, DC vaccines have shown good progress in anti-infection [22], organ transplantation, allergic diseases, and autoimmune diseases [23].

### 2.2. Peptide-Based Neoantigen Vaccines

Peptide vaccine platforms are by far the most common neoantigen vaccine platforms in clinical and early-stage studies. Peptide vaccines can be short peptide chains of approximately nine amino acids or long peptide chains of 20–30 amino acids. Unlike short peptide vaccines, long peptide-based neoantigen vaccines need to be endocytosed, processed by APC, and then bound to MHC molecules. The binding of long peptides to MHC II molecules activates CD4^+^ T cells [24]. Activating CD4^+^ T cells could assist in the specific initiation and function of CD8^+^ T cells.

Peptide vaccine platforms have been extensively used in cancer vaccines due to their ease of synthesis, low cost, and low toxicity. After sequencing and analyzing malignant and healthy cells by whole exome sequencing, Ott’s team designed a vaccine of up to 20 predicted individual tumor neoantigens—“NeoVax”—that induced multifunctional CD4^+^ and CD8^+^ T cells to target 58 (60%) and 15 (16%) of the 97 novel neoantigens found in tumor patients, respectively. In a follow-up of six vaccinated melanoma patients, it was found that the cancer did not relapse in four patients, and two relapsed patients also achieved tumor regression when combined with anti-PD-1 [25]. This not only proves that the neoantigen peptide vaccine induces neoantigen-specific T cells in vivo but also provides strong evidence that the combination of the vaccine with other means improves treatment. Neoantigen-based peptide vaccines are also proving attractive for treating nonsolid tumors such as glioma [26] and leukemia [27].

Toll-like receptor 2 (TLR2) is a pattern recognition receptor (PRR) whose ligands have a significant effect as adjuvants on stimulating immune responses. Peptide vaccines generally need to be used in combination with appropriate adjuvants to overcome the problem of poor immunogenicity. Thomas’s team designed an optimized couplable TLR2 ligand, mini-UPam, that couples to peptides and enhanced neoepitope-specific CD8^+^ T cell activation [28]. 

Antigenic peptide-based vaccines are safer, more stable, have lower antigenic complexity, and can carry multiple epitopes, unlike other vaccines [29]. However, peptide vaccines are susceptible to the limitations of immunological adjuvants and tend to be weakly immunogenic in the immunosuppressive tumor microenvironment; as a result, neoantigen peptide vaccines often face the challenges of vaccine adjuvant screening and combination strategies in oncology therapy [9]. In addition, the high heterogeneity of the MHC, the instability of the peptide chains in vivo, and even the timing and frequency of vaccination affect their use in clinical therapy [30].

### 2.3. RNA/DNA-Based Neoantigen Vaccines

RNA and DNA vaccines are all genetic vaccines based on exogenous gene replication coding information such as TSA or TAA on the surface of the tumor in the individual body to activate the immune response. As a critical research neighborhood in recent years, nucleic acid vaccines’ design and synthesis methods are convenient and highly safe.

The first successful mRNA transfection followed the discovery of mRNA transfection by Robert Malone in 1987 in frog embryos in vivo and the suggestion that “RNA could be used as a form of medicine” [31]. The advantages of RNA vaccines are that they do not insert into the host genome [32], avoid potential safety issues and allow for targeting multiple targets [33], have intrinsic adjuvant properties [34], and only need to be presented to the immune system through entry into the host cytoplasm, where their translational mechanisms are utilized to produce sufficient antigens. Rojas et al. utilized mRNA vaccines to treat pancreatic ductal adenocarcinoma (PDAC), inducing neoantigen-specific CD8^+^ T cells in eight patients, and expanding more specific T cells with boosters resulted in a longer median recurrence-free survival (at 18-month median follow-up) [35]. Over the last decade, there has also been some progress in investigating neoantigen-based RNA vaccines to treat gastrointestinal cancers [36], renal cancers [37], hepatocellular carcinomas [38], melanomas [39], and lung cancers [40].

To solve the problem of easy degradation and expression of RNA, RNA structure can be modified, such as through codon optimization [41], nucleotide modification [42], appropriate modification of structural elements (UTR’s, 5′ capping, ORF, Poly(A) tail) [43], or piggybacking with liposomes [44], nanosheets [45], and so on.

Unlike RNA vaccines, DNA vaccines must cross cellular and nuclear membrane barriers to enter the nucleus to be effective, and when delivered with the aid of electroporation, they are capable of generating high MHC class I affinity, driving CD8^+^ T cell immunity, and effectively generating antitumor effects [46]. Combining immunosuppressive agents can improve the DNA vaccine effects, and neoantigen DNA vaccines utilize erythrocyte capture to target the spleen and inhibit the growth of hepatocellular carcinoma. The antitumor efficacy was increased even further when combined with anti-PD-1 inhibitors, resulting in a 75% regression of subcutaneous tumors and a complete regression of in situ tumors, prolonging survival [47].

For the integration of multiple antigens, DNA vaccines are an efficient platform. Li et al. developed and optimized a DNA vaccine that targets multiple neoantigens in the E0771 and 4T1 models of mammary carcinoma in mice, as predicted by genome sequencing and computational methods. Most neoantigen-specific T cell responses could be induced after vaccination. Sustained immune responses were also achieved during vaccination with the anti-PD-L1 combination. Excellent results have also been shown in pancreatic cancer models [48].

There are several limitations to the development of nucleic vaccines: naked mRNA is degraded and inactivated by extracellular enzymes [49], inappropriate modification of RNA may reduce the effectiveness of the immune response [39], immunogenicity limits the applications of DNA vaccines, and there is a risk when integrating the DNA strand into the host gene. Other side effects include headache, fever, and pain or swelling at the injection site [50].

## 3. Whole Tumor Cell Vaccine (WTCV)

Whole tumor cell vaccines (WTCVs) are a type of cancer immunotherapy that uses the whole or lysed tumor cells, either intact or genetically modified, as a source of antigens and other immunogenicity factors that could stimulate an anticancer immune response. Due to the heterogeneity of tumors, targeting a single antigen can lead to select antigen-negative tumor cells, promoting tumor resistance and therapy failure. Therefore, targeting multiple antigens is a more competent solution. WTCVs contain the full spectrum of TAAs, which have important application prospects for inhibiting the occurrence, development, and recurrence of tumors [51]. In general, tumor cells obtained from patient tumors, animal orthotopic transplanted tumors, and laboratory-grown cell lines are subjected to physical, chemical, or biological processes to remove their tumorigenicity while preserving their immunogenicity. Moreover, due to their poor immunogenicity, tumor cells usually require surface modification, genetic engineering, immunogenicity death induction, and immunomodulators to increase their immunogenicity. After the vaccine has been injected into the body, APCs will take up, process, and present TAAs, recruiting and activating T cells to suppress tumors. WTCVs can be injected directly or loaded on DC cells. Depending on their source, tumor cells can be classified as autologous or allogeneic [52].

Autologous WTCVs, in which tumor cells are extracted from patients, prevent specific autoimmune responses from occurring due to MHC mismatch. Ye et al. evaluated rWTC-MBTA in breast cancer (4T1) and melanoma (B16-F10) models; the vaccine consisted of irradiated tumor cells modified with anti-CD40 antibodies, TLR agonists, and mannan-BAM. This vaccine enhanced activated T cells by increasing the proportion of APCs, effector cells, and memory cells, thereby delaying tumor growth and metastasis and prolonging overall survival [53].

However, when some patients’ tumors develop rapidly, or the complexity of the situation makes surgical sampling impossible, allogeneic WTCVs (allo-WTCVs) are more advantageous. It is easier to make and store allo-WTCVs in large quantities by extracting tumor cells of the same type from the allogeneic sources that possess the same tumor-associated antigens. Leaf et al. designed a hybrid tumor cell–dendritic cell vaccine (DCOne vaccine) for hematologic malignancies including multiple myeloma (MM). It was found that the specific antigens released by the DCOne vaccine were transported through its released extracellular vesicles (EVs), which activated APCs, induced activated T cells, and triggered CD8^+^ T cells to attack tumor cells [54].

WTCV development is limited by the difficulty in obtaining materials for auto-WTCVs, the immature preparation technology, the tedious and lengthy preparation process, the immunosuppression and immune escape of allo-WTCVs, and the possible heterogeneity [55].

## 4. Nanodelivery Platform for Vaccine

**Figure 2:**
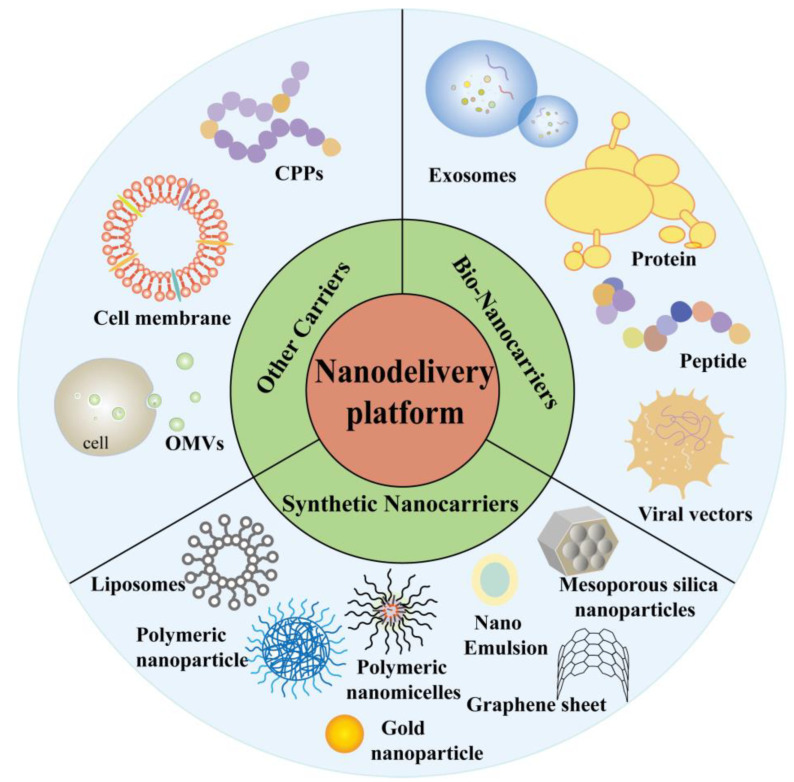
Nanodelivery system for tumor vaccines.

Conventional vaccine therapy suffers from the weak immune activation of DC vaccines, which requires repeated vaccinations [18], the susceptibility of peptide and nucleic acid vaccines to degradation, poor efficacy, and targeting, which are expected to be improved with the continuous development of nanotechnology [56]. The following section focuses on biological nanocarriers, liposomes, polymeric nanocarriers, and inorganic nanomaterials (Figure 2). Meanwhile, this paper lists a series of published nanodelivery vaccines for the treatment of various tumors (Table 1).

### 4.1. Bio-Nanocarriers

Biological nanocarriers are nanomaterials derived from organisms themselves. Some normal cells, tumor cells, tumor tissues, blood, and body fluids secrete exosomes or vesicles, as well as proteins and viruses, which may serve as biocompatible and low-toxic bio-nanocarriers.

#### 4.1.1. Exosomes

Exosomes are extracellular vesicles ranging from 30 to 100 nm in diameter, which were first identified in sheep reticulocytes in the 1980s. Major exosome components include cell-derived lipids, proteins, glycoconjugates, RNA, and DNA [99]. With increasing studies on the function and mechanisms of exosomes, it was found that in addition to being a way for cells to excrete waste products, exosomes are now also considered as specifically secreted membrane vesicles that can be involved in intercellular communication in various normal and pathological process [100]. Moreover, exosomes have been shown to act as antigen-presenting vesicles and therefore as stimulators of the immune response. Exosomes are natural nanocarriers. They have the advantages of low cytotoxicity, improved targeting, and enhanced immune response [101,102].

Many types of cells secrete exosomes, including immune and tumor cells. Tumor-derived exosomes (TEXs) exhibit a double-edged sword effect on the immune system, i.e., immunostimulatory or immunosuppressive effects. On the one hand, TEXs can promote cancer cell growth and invasion, expression of immunosuppressive molecules like PD-L1 and TGF-β, inhibition of CD8^+^ T cell activation and proliferation, etc. TEXs, on the other hand, express multiple MHC I molecules and markers of the tumor, like heat shock proteins (HSPs) engaged in antigen presentation and T cell stimulation, and are able to induce antitumor responses that are dependent on CD8^+^ T cells in vitro and in vivo. Moreover, TEXs act as a carrier of TAAs or TSAs to activate antigen-presenting cells (APC) like DC cells. Exosomes were used as a more potent antigen for DC loading compared to traditional tumor lysate. TEXs can be used as “mini-APCs” and as a new type of immunotherapeutic agent. Exomes may also be modified as adjuvants in nanovaccines. Autologous tumor lysate dendritic cell vaccine (ADC) has been shown to stimulate T cells with potential antitumor activity. ADC was able to induce tumor-specific T lymphocytes in a clinical phase III trial, was highly tolerated, and had an overall survival benefit in colorectal cancer patients [103]. In addition, it has been reported that tumor cells treated with pharmacological or ablative therapies, such as drug therapy and radiofrequency ablation (RFA), can induce an immune response within the tumor, promoting tumor antigen release and DC maturation [104]. Zhou et al. treated pancreatic cancer cells with mitoxantrone (MTX) to induce immunogenic cell death (ICD), producing mature exosomes (MEXO) used to modify the pancreatic cancer vaccine (spMEXO), which was able to enhance immune response as well as targeted delivery in vivo [105]. spMEXO showed higher cellular uptake than Lipofectamine 6000, indicating the potential delivery efficiency of exosomes. Liu et al. loaded serum exosomes (hEX) from hyperthermia tumor-bearing mice with black phosphorus quantum dots (BPQDs) and combined the vaccine with PTT action in another study, which had outstanding therapeutic effects in lung cancer treatment, such as more effective tumor targeting, PTT performance prolonging, and potent immune modulation [87].

DC-derived exosomes (Dex) carry MHC peptide complexes capable of directly or indirectly activating specific T cell immune responses [106]. Li’s team then designed a new antigenic vaccine delivery based on DC-derived exosomes. Neoantigen peptides M27 and M30 from melanoma and Adpgk, a colon cancer neoantigen, were electroporated into exosomes. The nanovaccine (Exo-) showed a “depot effect” at the site of administration by fluorescence signaling while enhancing lymphatic delivery and APC presentation without an excessive immune response. In comparison with liposomal vaccines (Lipo-), exosome-based vaccines show stronger tumor suppression and prolongation of survival because they carry proteins that can perform different functions [107]. Instead of electroporation, a “trigger” vaccine (DEX_P&A2&N_) can be formed simply by combining HCC-targeting peptide (P47-P) and pains DEX, an immune adjuvant that recruits DCs for activation. DEX_P&A2&N_ enhances the presentation of tumor neoantigens and activation of T cells, eradicating the HCC in the situ model of the mouse; combined with the FMS-like tyrosine kinase 3 ligand (Flt3L), it can remodel the immune microenvironment for a long-lasting antitumor effect [90].

Recent studies have also found that mouse serum-derived exosomes can also serve as a carrier for a neoantigen, and coating a neoantigen peptide from melanoma or colon cancer on serum exosomes can increase DC uptake and LN homing, expanding the immunogenicity of the antigenic peptide. Combined use with anti-PD-1 can completely eradicate tumors and achieve sustainable immunity [108]. In addition, CpG oligodeoxyribonucleotide (CpG ODN)-loaded cancer cell apoptotic bodies (Abs) secreted with macrophage-polarized exosomes to produce a series of cascade amplification [109], and the use of exosomes carrying both tumor and pathogenic antigens can also greatly improve immunogenicity [110].

#### 4.1.2. Protein/Peptide-Based Nanocarriers

Proteins and peptides have garnered much attention as nanocarriers because they are biocompatible, biodegradable, and easy to functionalize, which can increase the stability of drugs, peptides, and other substances, reduce their degradation rates [111], and improve the immunogenicity of vaccines and their drainage to lymph nodes [91].

Mehta’s team then attempted to optimize vaccine efficacy by fusing peptide epitopes to carrier proteins, which improved effective lymphatic uptake, protected the peptide epitopes from hydrolysis, and was shown by pharmacokinetics to elicit up to 90-fold immunogenicity [112]. Schmitt’s team fused the TLR5-binding D0/D1 structural domain of bacterial flagellar proteins (αCD40.Flg CMV) to the αCD40 antibody gene containing a neoantigen-derived peptide to form antibody–antigen coupler-adjuvant multifunctional antibody constructs capable of targeting DCs to induce specific T cell responses. Flagellar proteins are found in animal studies and clinical trials to induce antitumor [94] and antiviral [113] immune responses.

Ferritin is a self-assembled protein shell of 24 subunits and an iron core. Wang et al. have proposed to use SpyCatcher-modified ferritin nanoparticles to carry MC38 tumor-derived neoantigens. This NP vaccine was able to rapidly drain into lymph nodes, targeting dendritic cells, efficiently captured by DLN DCs in vivo, and eliciting efficient and specific CTL responses [58].

The ability of albumin to target tumors as a carrier for vaccines is because albumin can provide a large number of the nutrients needed for rapid cancer cell proliferation, allowing the presence of receptors on cancer cell surfaces that are capable of binding to albumin. Albumin as an endogenous substance can avoid the insecurity and complexity of some synthetic nanomaterials. Researchers have designed an albumin-binding vaccine (AlbiVax), and albumin/AlbiVax nanocomplexes can be delivered to LNs with high efficiency to induce effective and long-acting T cell responses. The delivery of neoantigen vaccines using albumin/AlbiVax nanocomplexes effectively enhances specific immunity and improves side effects. Combination with checkpoint inhibitors enhanced therapeutic efficacy [114].

Peptides have high biological activity, and the properties of their structures allow for various chemical modifications and possible targeting, making them promising for vaccine delivery. It was reported that DP7, a cationic hydrophilic novel antimicrobial peptide (AMP), modified with cholesterol has been found to have the dual role of delivery carrier and immunoadjuvant [89]. DP7-C can efficiently deliver a wide range of antigenic peptides to more than 75% of the DCs; at the same time, it can induce DC maturation and pro-inflammatory cytokine release through the TLR2-MyD88-NF-κB pathway, which improves antigen presentation efficiency and plays the role of an immune adjuvant. DP7-C/neoantigen-pulsed DC vaccines, consisting of direct incubation of DP7-C with neoantigens, effectively enhanced the antitumor efficacy of DC vaccination while promoting the MoDC uptake and presentation in advanced lung cancer patients.

DP7-C has also been validated to deliver microRNA involved in the remodeling of the tumor microenvironment. Its transfection efficiency and cytotoxicity were more advantageous compared to Lipo2000 and PEI25K [115]. In another study based on DP7, a simple dendrimer polypeptide (KK2DP7) nanoparticle was designed to improve the targeting of LNs, and in combination with immunosuppressants, it also prevented tumor recurrence [116]. This peptide-based delivery platform is also expected to be further developed for delivering peptide- and nucleic acid-based vaccines, especially for mRNA delivery, as negatively charged mRNAs are susceptible to electrostatic binding with cationic peptides [117].

#### 4.1.3. Virus (Virus-like Particle)/Bacterium-Based Nanocarriers

Immunogenic viruses or recombinant viruses as cancer vaccine vectors can stimulate tumor antigens to generate an immune response. Adenoviruses (Ads) are one of the most well-studied and promising viral vectors [118]. D’Alise et al. demonstrated that the gorilla Ad vaccine targeting tumor neoepitopes combined with αPD-1 in a mouse model could increase neoantigen-specific CD8^+^ T cells, improving immunogenicity and antitumor efficacy while potentially alleviating resistance to the PD-1 blockade [119]. 

Virus-like particles (VLPs) are hollow particles containing one or multiple viral structural proteins. VLPs have virus-like structures and properties, which can be effectively absorbed by APCs to induce immune responses; relatively, they are safer due to the absence of infectious genetic material, and they have the ability for autonomous replication [120]. Virus-like particles (VLP) extracted from phage P22 can be used as carriers of neoantigen peptides to design personalized therapeutic vaccines. Such vaccines can induce robust specific cellular and humoral immune responses and immune memory in vivo. Interestingly, this delivery platform can overcome the immunosuppressive microenvironment of the tumors [95]. VLPs have the advantages of clear composition, excellent biocompatibility, fixed spatial structure, and clinical translational potential.

Bacteria as a means of vaccine delivery has also attracted the attention of many researchers. ΔppGpp S. typhimurium has been found to highly target tumors, induce immune cell infiltration, and promote tumor suppression [121]. Hyun et al. then used Salmonella typhimurium, which has high tumor-infiltrating abilities, to piggyback on a neoantigen peptide that has high tumor-targeting abilities. A single injection revealed a large increase in CD4^+^/8^+^ T cells in mice, inhibition of tumor growth, and prolongation of survival [122].

At this stage, tumor immunotherapy with exosomes, proteins, or viruses as carriers shows significant biological advantages: good biocompatibility, low cytotoxicity, and alleviation of immune tolerance, and it is worthwhile to further explore the development of biological nanocarriers as a vaccine platform for tumor immunotherapy.

### 4.2. Lipid Nanoparticles (LNPs)

Liposomes are the first LNPs to appear and have a bilayer vesicle structure [123], consisting mainly of phospholipids and cholesterol, which form a solid framework that is amphiphilic. The incorporation of cholesterol reduces the fluidity of liposome membranes and the permeability of aqueous molecules, increasing the stability of lipids in body fluids. Since the 1990s with the introduction of doxorubicin (Doxil™) liposomes, the first nanomedicine for clinical oncology treatment, liposomal anticancer drugs have been now widely used in anticancer therapy [124], and liposomes can be used as a platform for delivering vaccines, releasing antigens in a controlled and slow manner, and inducing immune responses [125].

**Figure 3:**
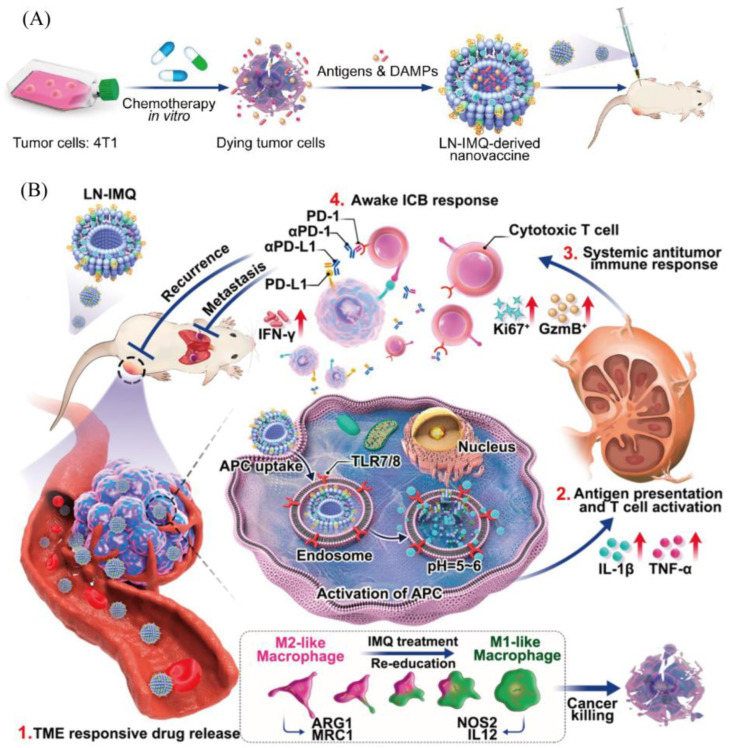
(**A**) LN-IMQ can encapsulate neoantigens induced by chemotherapeutic agents, with encapsulation rates up to 67.4%. (**B**) LN-IMQ-derived vaccines are able to trigger potent antitumor responses by modifying the tumor microenvironment in combination with ICB. Reprinted with permission from ref. [127]. Copyright 2023 American Chemical Society. Notes: red up arrow means activation or upward adjustment.

The shape, size, and surface charge of liposomes are important factors affecting immunoreactivity. Optimization of liposomes by changing the membrane composition and employing modifications with different moieties can be achieved to target vaccines actively or passively to different cellular and tissue sites. Modification of liposomes with a pH-sensitive polymer (ChexPG-PE) and a TLR2 ligand (h11c) can serve as a tool for neoantigen vaccine transport. The modified liposomal vaccine (h11c-Chex) enhanced the target activation of DCs and inhibited tumor growth [126]. The design of liposomal nanoparticle platforms responsive to the acidic tumor microenvironment enables controlled delivery and release of neoantigens. Flexible nanoplatform ratios also reduce systemic cytotoxicity while maintaining efficacy. Cancer immunotherapy response could be enhanced by combination with immune checkpoint blockers (Figure 3) [127].

In addition to using modifications to alter the properties of liposomes, researchers have also combined tumor vaccines and photothermal therapy or ICIs to improve antitumor efficacy. Cuixia Zheng et al. prepared an FA-TSL/AuNCs/SV nanoplatform by integrating folic acid-modified thermal-sensitive liposomes (FA-TSL) as the shell and simvastatin (SV) adjuvant-loaded Au nanocages (AuNCs) as the cores. The liposomes can be engulfed by tumor cells more effectively due to FA decoration. Tumor-derived protein antigens (TDPAs) were released due to photothermal treatment (PTT) mediated by AuNCs, which was further captured by AuNCs/SV, and formed the in situ recombinant vaccine (AuNCs/SV/TDPAs). AuNCs/SV/TDPAs could efficiently transport to draining LNs owing to the hyperthermia-induced vasodilation effect and small particle size, achieving co-delivery of antigens and adjuvant for the initiation of specific T cell response [77]. In a study, a colon cancer cell-derived neoantigen peptide, Adpgk, was encapsulated into liposomes (Adpgk-BPQDs-liposome) along with black phosphorus quantum dots, and the combination of ICIs blocked tumor progression [57].

Lipoproteins are a class of particles that act as transporters in the bloodstream in vivo and can be categorized mainly into high-density lipoproteins and low-density lipoproteins. Lipoproteins are a highly desirable delivery platform, highly biocompatible, and suitable for modification in a variety of ways to target specific tissues [128]. Synthetic high-density lipoprotein (sHDL) presents a promising future as a new liposome-based platform in neoantigen delivery systems. Kuai et al. developed nanodiscs of sHDL based on phospholipids and ApoA1 mimetic peptides. sHDL is highly secure, has multiple loading sites, and is very small in size (~10 nm) [96]. Cholesterol-CpG and neoantigen peptides are extremely easy to assemble into sHDL nanodiscs, presenting a homogeneous, stable, and ultra-small state. This tumor vaccine promotes Adpgk/CpG presentation to APCs, activates broad-spectrum T cell responses, and eradicates tumors in combination with ICI, and neoantigen Adpgk-based nanodiscs also produce significant immune responses in mouse models [129].

Cationic lipids are another type of lipid nanoparticle with greater space and greater potential for vaccine loading. The cholesterol-modified cationic peptide DP7 (DP7-C) has been shown to act as an immune adjuvant and delivery vehicle to stimulate DC maturation and enhance immune responses to neoantigen pulsed DC vaccines but is unable to load mRNA. However, after using DOTAP liposomes, it was found that mRNAs were successfully piggybacked on liposomes, and the neoantigen vaccine (DOTAP/DP7-C/LL2 neoantigen mRNA complexes) modified with DP7-C significantly improved the transfection efficiency and antitumor effect of introducing mRNAs encoding neoantigens into DCs, and it promoted DC maturation and lymphocyte-specific responses compared to DOTAP liposomes [83].

Cationic lipids contain ionizable (cationic) head groups. These form stable complexes with negatively charged and hydrophilic nucleic acids, and after endocytosis into the cell, intracellular anionic lipids neutralize the carrier charge so that the negatively charged nucleic acids are released. Cationic lipids are the most extensively used nonviral system for the delivery of nucleic acids, but it is worth noting that their effectiveness and toxicity are correlated, with multivalent cationic lipids being more effective but more toxic than the monovalent state, and that side effects may be reduced if they are similar in structure to naturally occurring lipids (e.g., cationic cholesterol) [130]. A negatively charged DNA vaccine encoding a neoantigen (DD-TMG-IL12/CpG) can bind to cationic lipids by electrostatic interaction to form a more stable cationic lipid–nucleic acid complex, reducing nuclease degradation, facilitating systemic delivery of the vaccine, and markedly inhibiting melanoma tumor growth and lung metastasis in mice with less cytotoxicity [79].

Although TSA is an ideal target for direct targeting of cancer cells, the long time required to identify neoantigens and the complexity of the technique makes direct targeting limited. Yu et al. proposed a whole tumor cell lipid nanovaccine targeting LN, α-melittin-NP, which utilizes melittin properties to directly induce tumor cell death and help the release of whole tumor antigens in situ. The modified melittin reduced the toxicity to erythrocytes while possessing the optimal size for targeting LNs and the activation ability of APCs [131].

LNPs have significant potential in delivering nucleic acids and peptide chains [125,132], preventing nucleases, avoiding endosomal capture and phagocytosis, and promoting cellular uptake [133]. In addition to cationic lipids, the development of new-generation LNPs like solid lipid nanoparticles [134], immunoliposomes [135], and nanostructured lipid carriers [136] has solved some of the previous liposome’s deficiencies: enhanced long-term physical stability, loading capacity, and bioavailability. In addition to the advantages, issues such as the capture efficiency of LNPs in vivo and the actual cost of production need to be further considered.

### 4.3. Polymeric Nanocarriers

**Figure 4:**
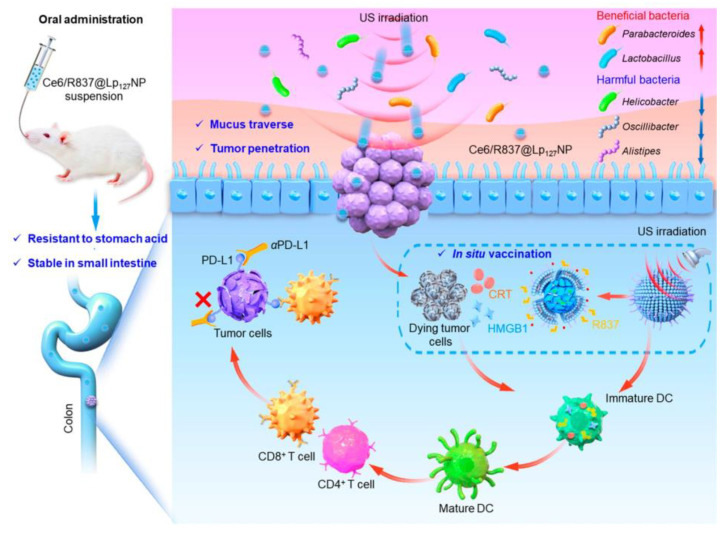
After oral administration, the nanoparticles stably reach the gastrointestinal site, successfully traverse the mucosal barrier, and aggregate at the tumor site under US irradiation, inducing the ICD of tumor cells, releasing a large number of antigens, and R827 synergizes with the neoantigens. Reprinted with permission from ref. [138]. Copyright 2024 American Chemical Society. Notes: red up arrow means activation or upward adjustment; blue down arrow means decrease; checkmark means what nanoparticles can achieve.

Polymeric vectors are a class of systems with a large range of sizes (10–1000 nm); they are mainly natural polymers or synthetic polymeric materials loaded with antigens and immune adjuvants by adsorption, coupling, or covalent binding, in which polymers rich in side chains can achieve multifunctional loading, and they target LNs or tumor sites by controlling their size, hydrophobicity/hydrophilicity, and charge [137]. The design concept of these polymeric vectors provides a new idea for in situ tumor vaccine research. A personalized vaccine for the treatment of colorectal cancer based on an oral modality for in situ vaccination has been identified in a new study. Ce6/R837@Lp127NPs are based on silk fibroin nanomaterials with an immunoadjuvant (imiquimod, R837) and a sonosensitizer (chlorin e6, Ce6), coated with Pluronic F127 (p127) and natural lipids to ensure that they can overcome the obstacles of the mucus barrier. It can induce colorectal cancer cell death by sonodynamic therapy, and the generated neoantigens in the presence of R837 promote DC maturation, sustained tumor suppression, and in situ vaccination (Figure 4) [138]. This provides a novel and reliable idea and a means of treating CRC in situ.

Polyethyleneimine (PEI) is a cationic polymer, a synthetic polymeric nanomaterial, widely used for antigen vaccine delivery. Electrostatic self-assembly of PEI-coupled neoantigens with CpG adjuvant into nanocondensates promotes cross-presentation of antigens and neoantigen-specific CD8^+^ T cell activation, providing a simple and efficient delivery platform for neoantigen vaccines [139]. In addition to nanoparticles, PEI self-assembled with farnesyl thiosalicylic acid to form amphiphilic micelles (FTS-PEI) was able to be more efficient in tumor transfection and mediate neoantigen vaccine vaccination compared to PEI and its derivatives [140].

For the delivery of mRNA, it is necessary to ensure that it can be protected from enzymatic degradation or directly transported to the cytoplasm or lysosome for escape. It has been found that fluorinated compounds with hydrophobic and lipophobic amphiphilic properties can penetrate the lipid bilayer of the cell membrane and the lysosomal membrane [141]. After modification of PEI with fluorinated compounds, the nanovaccine (F-PEI/mRNA) formed by self-assembly with the neoantigen may have the possibility of lysosomal escape for efficient delivery of mRNA. As a result, F-PEI/mRNA was found to induce higher levels of MHC I antigen presentation and robust specific immune responses [142]. Gong et al. made a very attractive proton-driven nanotransformer-based vaccine (NTV) based on the properties of different polymeric materials, where self-assembled spherical nanostructures dissociate and reassemble themselves into nanofibers and nanosheets in acidic medium, disrupting the in vivo membrane to release neoantigens directly into the cytoplasm [67].

Natural materials such as glucans and chitosans have higher safety profiles compared to synthetic polymeric materials. Saccharomyces cerevisiae β-1,3-glucan particles (GPs) coupled with peptides can form a new antigenic vaccine delivery system (GP-Neoantigen) with highly targeted APCs. In addition, this vaccine system is more stable between batches and has a uniform particle size compared to other synthetic nanoparticles. Significant immune infiltration was detected in mice after vaccination with a vaccine loaded with breast cancer neoantigen, inducing specific T cell immune responses and humoral immunity [68]. Chitosan nanoparticle vaccines loaded with whole tumor cell lysates have also been found to target specific DCs, and in vivo studies in mice have found that the nanovaccines can be taken up by endogenous DCs, eliciting cellular immune responses [143]. Overall, these studies have illustrated the ability of polymeric nanoparticles to serve as a self-assembling, excellently flexible, and highly efficient delivery platform for new antigenic vaccines with advantages such as lysosomal escape, targeting of LNs, uptake by APCs, and enhancement of cellular immune responses.

Polymeric materials can also be delivered in the form of depots: hydrogels, microcapsules, microneedles, etc. A hydrogel is defined as a 3D network based on polymer chains [144], which has some degree of similarity to biological tissues, and has a broad range of applications in wound recovery and drug/antigen delivery. Delitto et al. found that implanting a hydrogel vaccine (PancVax) with pancreatic cancer neoantigens into the PDAC resection site exerted a long-lasting effect, inducing immune infiltration and immune cell mass activation, preventing tumor recurrence while promoting wound healing [98]. This is a new approach to the prevention of tumor recurrence and improvement of prognosis in the clinic. The idea of using a rich variety of nanoparticles in combination with hydrogels to solve the problem of limited types of hydrogel-loaded substances has aroused extensive attention. A thermosensitive hydrogel carrying black phosphorus quantum dot nanovesicles (BPQD-CCNVs) encapsulating tumor cell membranes was prepared by Ye et al., containing lipopolysaccharide (LPS) and granulocyte-macrophage colony-stimulating factor (GM-CSF). This personalized photo-thermal hydrogel vaccine has both the slow-release effect of the hydrogel and the local recruitment and antitumor effect of the BPQD-CCNVs nanoparticles, which avoids repeated injections and improves the uptake of the vaccine [82]. Additionally, hydrogels (Gel) containing bacterial-derived vesicles loaded with neoantigens and GM-CSF can continuously recruit DCs [145]. A PDT-driven autologous tumor cell vaccine (P-ATV) coated by PEI-Ce6 with a Fmoc-KCRGDK-phenylboronic acid (FK-PBA) hydrogel targeted tumor cells that overexpress sialic acid, and the gel was injected serially on demand in the area of residual tumor [80].

Unlike hydrophilic hydrogels, microcapsules can be loaded with diverse substances, building a plentiful antigen library. Xi reported a self-healing microcapsule capable of carrying protein/peptide chains, which modulates the immune microenvironment in situ, effectively loading antigen molecules into macroporous microspheres based on polylactic acid (PLA) and poly(ethylene glycol)-b-poly-dl-lactide (PELA) to form a sustained release antigen library, promoting the uptake and cross-presentation of the antigen and recruitment and activation of APCs [146]. In addition, polymeric microneedle vaccines based on transdermal delivery have been explored, for instance, biodegradable microneedles (bMNs) based on PEG- and PSMEU-carrying DNA vaccines can elicit specific humoral immune responses and continuously release and inhibit lung metastasis [147]. Another is a patch of B16F10 whole tumor lysates loaded into a polymer microneedle that releases the lysates continuously for 5 days after insertion into the skin, and under NIR light irradiation, the patch’s self-contained melanin produces heat, which promotes the uptake of antigens by DCs and enhances antitumor vaccination [148].

### 4.4. Inorganic Nanocarriers

In addition to these natural or synthetic organic materials mentioned above, inorganic materials have also attracted attention due to their diverse and stable structures, unique optical and electrical properties, and biocompatibility. These inorganic materials with special physicochemical properties are mainly loaded with antigens through covalent bonding, electrostatic adsorption, and coupling, pioneering new modes of vaccine delivery [137] and providing a platform that can be combined with photothermal therapy [149], photodynamic therapy [59], and magnetic resonance imaging [150], among other methods. Common inorganic nanomaterials include mesoporous silica, carbonaceous materials, and metal/metal compound nanoparticles.

Mesoporous silica is a porous material with ordered mesoporous molecular sieves, presenting properties that include a large specific surface area, stronger adsorption, higher biocompatibility, and lower cytotoxicity [151]. Therefore, mesoporous silica has great advantages in delivering drugs and vaccines and is one of the most stable and promising inorganic materials. Mesoporous silica nanoparticles (MSNs) are one of the more common forms. Mooney’s team developed a simplified and high-performance multi-antigen platform to enhance antigenic immunogenicity by using PEI in MSN vaccines. MSN-PEI vaccines containing neoantigen peptides were able to enhance DC activation and T cell responses, control tumor growth, and eradicate lung metastases [69]. To solve the problem of low efficiency of nanoparticle delivery to lymph nodes (dLN), Kim’s team also skillfully coupled MSN and mesoporous silica microrods (MSRs) to make an injectable dual-scale mesoporous silica vaccine, which formed a 3D macroporous scaffold capable of recruiting rich DCs after injection and generating a greater number of antigen-specific T cells compared to a single MSN or MSR vaccine to inhibit melanoma growth, providing a new platform for DC-targeted nanovaccines [152]. In addition, MSR has been shown to encapsulate p-DNA encoding multiple antigens, especially neoantigens, directly transfecting host DCs in scaffolds, overcoming the limitations of subcutaneous and intramuscular administration of DNA vaccines, and eliciting both cellular and humoral immune responses [153]. The combination of several neoantigen peptides, a CpG oligodeoxynucleotide adjuvant, and the photosensitizer chlorin e6 in biodegradable MSN (bMSN) is another more classic example. PDT and immune reactions act simultaneously, triggering the activation of neoantigen-specific T-lymphocytes, and simultaneous imaging combined with PDT immunotherapy can be used to treat advanced cancers [59].

In addition to rod-like structures, hollow nanoparticles are preferred for vaccine delivery. Zhu’s team constructed a thin-shelled hollow-structured PEI-HMSN, which effectively improved the antigen loading rate as well as sustained release [154]. Moon’s team encapsulated manganese oxide in hollow MSN nanoparticles (MnOx@HMSN), which released cyclic dinucleotides (CDNs); the manganese ions significantly enhanced STING activation and promoted DC activation and cross-presentation of neoantigen peptides. Vaccination with SARS-CoV-2 virus vaccine elicits a robust and long-lasting (approximately up to one year) humoral immune response [60]. However, the potential toxicity and complex modification process present in MSNs, as well as the high cost of synthesis, may be potential issues limiting the clinical translation of MSNs.

The carbonaceous material graphene oxide has become a hot spot for drug or vaccine delivery due to its unique physicochemical properties. Through electrostatic, adsorption, and coupling, graphene oxide sheets can achieve high multilayered antigen loading while preferring the folded state in APCs [155]. Moon’s team designed a highly modular, biodegradable PEGylated reduced graphene oxide nanosheet (RGO-PEG), with a diameter of around 20-30 nm, which can rapidly produce neoantigen vaccines. In targeting LN, RGO-PEG has the advantages of high efficiency and long-lasting accumulation. More notably, RGO-PEG generates reactive oxygen species in DC, and a single injection of RGO-PEG vaccine can induce a potent neoantigen-specific T cell immune response for up to 30 days [61]. Meanwhile, graphene oxide has self-adjuvant properties that can be modified to ameliorate the problem of nondegradability of carbonaceous materials. However, the biosafety experiments that need to be performed for each GO are also a burdensome task.

Magnetic particles, exemplified by iron oxides, have attracted widespread attention. The most important feature of magnetic particles is that they are inherently magnetic and visible with imaging, so they can not only be used for targeting purposes by magnetic actuation but also can be combined with magnetic resonance imaging (MRI) for guidance and evaluation of the effect. Based on magnetic iron oxide, Chen et al. made a dual-ligand nanoprobe (Fe_3_O_4_@RGD@GLU), which greatly increased the targeting of tumors by the addition of an external magnetic field while inducing local thermotherapy of tumors [156]. MRI guided the membrane-encapsulated magnetic nanoclusters (MNCs) of Fe_3_O_4_ in cancer cells to accurately target LNs, demonstrating a greatly prolonged retention time in the LNs and promoting MHC I cross-presentation [150].

Metal/metal compound nanoparticles are suitable for development as carriers of antigens. More widely used are gold nanoparticles (AuNPs), which can be made into a variety of shapes, including spheres, rods, and cubes, because of their stable nature and good ductility with a large specific surface area [157]. New delivery platforms (HSA@AuNPs) are made of human serum proteins (HSAs) that can be penetrated by the in vivo barriers combined with gold nanorods, which promises to efficiently combine localized photothermal therapy and immune response for antitumor therapy [149]. CuS nanoparticles, which also have high photothermal effects, were combined with DLMSNs to make AM@DLMSN@CuS/R848, which targeted TNBC, while laser-generated photothermal ablation promoted vaccine effects [158]. It has been proposed that the particle size and shape of metal nanoparticles tend to affect their ability to act as immune adjuvants. For carriers that cannot function as adjuvants or photosensitizers, it is reasonable to carry either adjuvants or photosensitizers. ZnP nanoparticles loaded with photosensitizer pyrolipid combined with photodynamic therapy (PDT) increased tumor immunogenicity [159]. Metal-based nanoparticles such as metallic aluminum [160], manganese ions (Mn^4+^) [84], MnO_2_ [161,162], CoO [163], and others can also be considered as potential neoantigen delivery platforms.

In recent years, metal–organic frameworks (MOFs, ZIFs) have been investigated as a new strategy for drug and vaccine delivery owing to their high surface area, high loading capacity, and adjustable pore size. These materials are usually pH-responsive [164] and reduction-responsive [165]. Zhong et al. designed aluminum-containing nano ZIF-8 particles (ZANPs) to deliver antigens and found that they could achieve a high antigen loading of 30.6% and dissociate in an acidic environment to release antigens [164]. Another novel mechanism is the oxidation nanoprocessing (AONP) strategy, based on the activation of peroxomonosulfate by ZIF-67 nanocatalysts to continuously generate SO_4_^−^ radicals, causing continuous oxidative damage to tumor cells, maintaining the cancer cell integrity and tumor antigen diversity [166]. The MOF structure has been found to contribute to the occurrence of lysosomal escape [167] and act as a photosensitizer [168].

### 4.5. Other Carriers

Biomimetic nanomaterials have multifunctionality and high biocompatibility. Biomimetic nanoparticles encapsulated by cell membranes can improve their stability in vivo [169]. The main sources of cell membranes include erythrocytes, leukocytes, cancer cells, and stem cells. Cancer cell membrane-encapsulated nanomaterials can provide tumor antigens and targeting ability to generate immune escape [170], and Padler-Karavani’s team applied erythrocyte membranes to an active cancer vaccine targeting Neu5Gc-TACAs, which made the vaccine biocompatible and prolonged circulation time [97]. Outer membrane vesicles (OMVs), formed mainly by shedding of Gram-negative bacterial cell membranes or portions thereof, are rich in proteins, polysaccharides, and nucleic acids; they are most qualified as immune adjuvants and can quickly display antigens [66,171].

Cell-penetrating peptides (CPPs) are virus-derived peptides, which can be broadly classified into cationic peptides, amphiphilic peptides, and hydrophobic peptides. CPP can effectively transport small-molecule drugs, proteins, peptides, and nucleic acid fragments through the cell membrane barrier into the cell using covalent linkage, electrostatic interaction, or hydrophobic interaction [172]. The nonviral vector CPP can transport nucleic acids by electrostatic interaction, avoiding adverse reactions caused by virus delivery. By modifying the peptide chain H5-S4(13)-PV with histidine at the N-terminus, DNA can be more easily delivered into the cell and has lower toxicity [173].

## 5. Outlook

Immunotherapy based on neoplastic tumor antigens can improve the central–peripheral tolerance response caused by conventional immunotherapy. Precision therapy targeting antigens unique to tumor cells reduces damage to nontumor sites, prolongs the action of T cells, and induces durable immune responses. Although immunotherapy based on nascent tumor antigens is more effective, it is challenging because tumor-specific expression of antigen genes is more difficult to detect, and most mutant antigens are still highly variable between individuals. In addition, the delivery system is one of the key components in maintaining the ability of the antigen to remain active and to be delivered efficiently, although the efficacy of a vaccine is entirely dependent on the properties of the antigen. Numerous studies have demonstrated that the discovery and rational selection of novel nanodelivery materials can effectively improve the stability and efficient delivery of neoantigens. Therefore, the collaboration between nanotechnology and tumor vaccines accelerates the development of neoantigen vaccines for tumors and creates an effective means of combining immunotherapy with different therapeutic modalities, which benefit tumor patients.

A combination of factors, such as treatment cost, patient health status, and readiness, must be considered before clinical application due to the high cost and time delay of individualized vaccines and the uncertainty of the optimal neoantigen discovery platform. Although there are many limitations in the direction of neoantigen vaccines for tumors at this stage, in the future, with the development of technology, we can find more and more suitable vectors to conduct clinical studies of personalized vaccines and provide more effective and safe treatments for tumor patients.

## Figures and Tables

**Table 1:** A brief summary of nanodelivered neoantigen vaccines for cancer treatment.

Types of Cancer	Vaccine Types	Vaccine Name	Composition	Combination with	Ref.
Colon cancer	peptides	Adpgk-BPQDs-liposome	liposome	PTT	[57]
		ferritin-E7 (43–62) NP/ferritin-Reps1/Adpgk/Dpagt1 NP	SC-ferritin NP	ICIs	[58]
		bMSN (CpG/Ce6)-Adpgk	bMSN	PDT	[59]
		MnOx@HMSN (CDA + Adpgk)	MnOx@HMSN	—	[60]
		RGO(CpG)-PEG-Adpgk	RGO-PEG	ICIs	[61]
		NP Vacc	PEI-PEG	STING agonist	[62]
		nanoSTING-peptide antigens-cGAMP	nanoSTING-vax	ICIs	[63]
		CNPs-TCL/neoantigen	CNPs	—	[64]
	RNA	RNA-LPX	liposome	CPI	[44]
		Nucl-TAP siRNA	nucleolin aptamer	ICIs	[65]
	protein	CC-SpT-Adpgk OMVs	OMV	—	[66]
Melanoma	peptides	NP Vacc	PEI-PEG	STING agonist	[62]
		nanoSTING-peptide antigens-cGAMP	nanoSTING-vax	ICIs	[63]
		NTV	NT2	ICIs	[67]
		GP-M30	GPs	—	[68]
		MSR-PEI E7	PEI	—	[69]
		AuNP@B16F10	AuNP	PTT	[70]
		PeptiCRAd-SIINFEKL	OVs	—	[71]
		EVX-01	cationic liposome (CAF@09b)	CPI	[72]
		DSPE-PEG2000-peptide-NIR797 nanoparticles	DSPE-PEG2000	ICIs	[73]
		peptide/pPAA nanoplexes	polyanion pPAA	—	[74]
		multi-target VLP-based vaccine (MTV)	Qβ-VLP/CpG	—	[75]
		M-NP-Ag	PLGA nanoparticles	DCV	[76]
	protein	FA-TSL/AuNCs/SV	AuNCs	PTT	[77]
	RNA	LNP-mRNA	LNPs	CpG2018B	[78]
	DNA	DD-TMG-IL12/CpG	liposome	—	[79]
	WTCV	PC-Cell@gel	FK-PBA hydrogel	PDT	[80]
		CpG@LL-B16F10	CpG@eBSA NPs	PDT	[81]
	tumor cell membrane	Gel-BPQD-CCNVs	BPQD-CCNVs	ICB, PTT	[82]
Kidney cancer	RNA	DOTAP/DP7-C/mRNA	DOTAP liposome	—	[83]
Cervical cancer	peptide	ferritin-E7 (43–62) NP/ferritin-Reps1/Adpgk/Dpagt1 NP	SC-ferritin NP	ICIs	[58]
		Mn^4+^-SNPs + GF001	Mn^4+^-SNPs	—	[84]
		cGAMP/antigen-codelivering NVs	NP	ICB	[85]
Breast cancer	peptide	GP-M25	GPs	—	[68]
		M-NP-Ag	PLGA nanoparticles	DCV	[76]
	RNA	CM-RNA@Ce6/PLGA	Ce6/PLGA	USI	[86]
Lung cancer	—	hEX@BP	hEX	PTT	[87]
	peptide	neoantigen peptide DSPE-PEG2000-NHS	DSPE-PEG2000-NHS	—	[88]
	DC	DP7-C/neoantigen-pulsed DCs vaccine	DP7-C	—	[89]
Hepatocellular carcinoma	peptide	DEX_P&A2&N_	DEX	—	[90]
		NGC-gels vaccines	silk gels	TIM-3 blockade	[91]
		thiolated nanovaccine	CpG-ODN NPs	ICB	[92]
		mD@cSMN	cSMN	PDT	[93]
AML	DC	αCD40-DC	bacterial flagellin	—	[94]
Lymphoma	peptide	VLP-neoantigen peptide	P22 VLPs	—	[95]
Glioma	peptide	NeoAgs-CpG-nanodisc	sHDL nanodiscs	ICIs	[96]
Neu5Gc-positive tumor cells	Neu5Gc-neoantigens	biomimetic glyconanoparticle vaccine	NGs	—	[97]
Pancreatic adenocarcinoma	peptide	PancVax gel	hydrogel	operation	[98]

Abbreviations: PTT: photothermal therapy; BPQDs: black phosphorus quantum dots; SC-ferritin NP: SpyCatcher-modified ferritin nanoparticle platform; ICIs: immune checkpoint inhibitors; sHDL: synthetic high-density lipoprotein; bMSN: biodegradable mesoporous silica nanoparticles; PDT: photodynamic therapy; HMSN: hollow MSN; RGO-PEG: PEGylated reduced graphene oxide nanosheet; PEG: polyethylene glycol; PEI: poly(ethylene imine); CNPs: CpG ODN-loaded nanocomplexes; TCL: tumor cell lysates; RNA-LPX: lipoplex-formulated RNA; CPI: checkpoint inhibitors; Nucl: nucleolin aptamer; OMVs: outer membrane vesicles; FA-TSL: folic acid modified thermal-sensitive liposomes; SV: simvastatin; NTV: nanotransformer-based vaccine; GPs: β-1,3-glucan particles; NP: nanoparticles; OVs: oncolytic viruses; DSPE-PEG2000: distearoyl phosphoethanolamine-PEG2000; pPAA: poly(propylacrylic acid); LNPs: lipid nanoparticles; PC-Cell: PEI-Ce6-coated autologous tumor cells; FK-PBA: Fmoc-KCRGDK–phenylboronic acid; eBSA: ethylenediamine-modified bovine serum albumin; BPQD-CCNVs: tumor cell membrane-coated black phosphorus quantum dot nanovesicles; Mn^4+^-SNPs: Mn^4+^-doped silica nanoparticles; cGAMP: cyclic GMP-GMP; hEX: exosomes from hyperthermia-treated mice; NHS: N-hydroxysuccinimidyl; DEX: DC-derived exosomes; cSMN: SiPCCl2-hybridized mesoporous silica with coordination of Fe(III)-captopril; VLPs: virus-like particles. Note: “—” means not mentioned in the text.

## Data Availability

Data is contained within the article.
